# Hepatoprotective Effect of Baicalein Against Acetaminophen-Induced Acute Liver Injury in Mice

**DOI:** 10.3390/molecules24010131

**Published:** 2018-12-31

**Authors:** Hong-Chao Zhou, Hui Wang, Kun Shi, Jian-Ming Li, Ying Zong, Rui Du

**Affiliations:** College of Chinese Medicinal Materials, Jilin Agricultural University, Changchun 130118, China; zhouhongchao813716@126.com (H.-C.Z.); wanghui813716@126.com (H.W.); sk1981521@jlau.edu.cn (K.S.); lijianming4773@163.com (J.-M.L.)

**Keywords:** baicalein, acetaminophen, liver injury, inflammation, autophagy

## Abstract

Baicalein (BAI), one of the main components of *Scutellaria baicalensis Georgi*, possesses numerous pharmacological properties, including anti-cancer, anti-oxidative, anti-virus and anti-bacterial activities. The purpose of this study was to evaluate the hepatoprotective effect of baicalein against acetaminophen (APAP)-exposed liver injury in mice, and elucidate the underlying hepatoprotective mechanism. Baicalein pretreatment significantly alleviated the elevation of IL-6, IL-1β and TNF-α in serum and hepatic in a dose-dependent manner. It also dose-dependently reduced the hepatic malondialdehyde (MDA) concentration, as well as the depletion of hepatic superoxide dismutase (SOD), hepatic glutathione (GSH) and hepatic catalase (CAT). Moreover, pretreatment with baicalein significantly ameliorated APAP-exposed liver damage and histological hepatocyte changes. Baicalein also relieved APAP-induced autophagy by regulating AKT/mTOR pathway, LC3B and P62 expression. Furthermore, the hepatoprotective effect of baicalein to APAP-induced liver injury involved in Jak2/Stat3 and MAPK signaling pathway. Taken together, our findings suggested that baicalein exhibits the ability to prevent liver from APAP-induced liver injury and provided an underlying molecular basis for potential applications of baicalein to cure liver injuries.

## 1. Introduction

Adverse drug reactions (ADRs) are a significant cause of illness and death in the world [1]. Drug-induced liver injury (DILI) is one of the serious ADRs which is an important clinical problem in the practice of hepatology [2]. DILI is caused by the biological activation of chemically active metabolites mediated by enzymes, which can lead to cell death and possible liver failure [1]. The particularity and poor prognosis of DILI make it a major safety issue in the process of drug development and also result in a serious clinical and financial problem [3]. It has been recognized that oxidative stress, sterile inflammation and compensatory liver repair and regeneration were key signaling pathways during the development of DILI [4]. Therefore, the inhibitions of inflammation, autophagy and oxidative stress might be considered as the potential strategies for the prevention and treatment of DILI.

Acetaminophen (APAP) is one of the most widely used antipyretic and analgesic drugs. It was recently reported that the APAP mediations were used by more than 60 million Americans on a weekly basis [5]. However, APAP overdoses can cause severe liver injury and even acute liver failure (ALF) and it has been widely used to induce acute liver damage in animal models for testing of hepato-protective interventions [6,7,8,9,10]. APAP-induced liver injury is not directly caused by the drug itself, but through the formation of the toxic metabolite N-acetylquinone imine (NAPQI), generated through the activity of liver cytochrome P450 (CYP450) system [11]. Excessive NAPQI depletes GSH, resulting in the covalent binding of cysteine, especially mitochondrial proteins when APAP overdose [4,12]. This could cause oxidative stress and dysfunction of mitochondria, thereby inducing hepatic necrosis [13]. N-acetylcysteine (NAC) is recommended by the U.S. Food and Drug Administration as the only currently available treatment for APAP overdose requires a complicated infusion regimen and has a high incidence of adverse events, including anaphylactic reactions [14,15]. Hence, the development of effective and low side-effect drugs is clearly needed.

**Figure 1:**
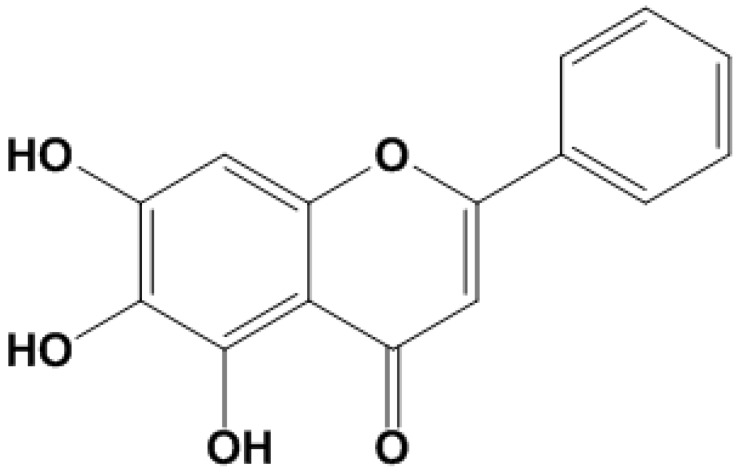
Structural formula of BAI.

The underground part of *Scutellaria baicalensis Georgi* (Huang Qin), which is obtained from several East Asian countries and the Russian Federation, and is now cultivated in many countries [16], has been widely used as a medicinal planet in China for thousands of years [17] for treating diarrhea, lung infections, liver problems and inflammation for a long time [18]. The pharmacological activities of *Scutellaria baicalensis Georgi* have been attributed mainly to its high flavonoid content. Baicalein (BAI, Figure 1) isolated from the roots of *Scutellaria baicalensis Georgi* is considered as one of the key active and characteristic compounds of Huang Qin. BAI has received rather more research attention because of its various biological activities including, anti-oxidative stress [19], anti-inflammatory [20], and anti-cancer properties [21]. BAI can regulate various complex signaling pathways to therapies disease. Yin et al. have suggested that baicalein could reduce inflammation in diabetic db/db mice via nuclear factor-κB (NF-κB) [22]. In addition, He et al. reported that baicalein, when co-administered with LY294002, could inhibit liver cancer cell proliferation and promote cell apoptosis by regulating PI3K/AKT signaling pathway [23]. In Xu’s report [24], it was revealed that the expression of signal transducer and activator of transcription (STAT) gene in the tyrosine protein kinase JAK/STAT signaling pathway in T cells could be downregulated by baicalein. Previous studies showed that BAI may have a protective role against APAP-induced hepatotoxicity. 

In the present study, the protective effects of baicalein on APAP-induced liver injury were investigated and the underlying molecular mechanisms were explored to develop an effective therapeutic agent to protect against APAP-induced liver injury.

## 2. Result

### 2.1. Effects of BAI on Body Weight and Organ Index in Mice

The changes of body weights before and after the experiment in mice and the organ coefficients of the liver and kidney were determined. 

As shown in the Table 1, APAP-induced mice gained less weight than the normal control. Liver and kidney coefficients were significantly increased following APAP administration (*p* < 0.05). However, the growth of liver weight and kidneys were significantly inhibited in the BAI administration group. 

### 2.2. Effects of Baicalein on APAP-Induced Liver Injury

**Figure 2:**
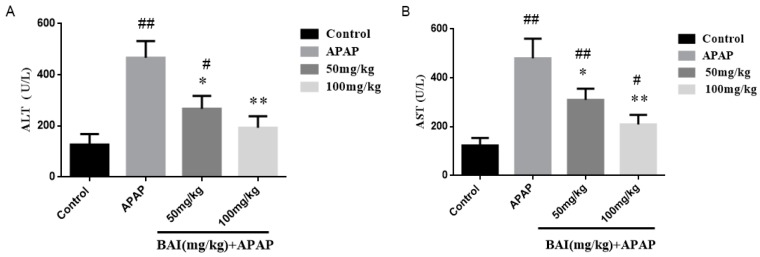
Effects of BAI on serum enzyme activity. 1h after the last dose of BAI, APAP (350 mg/kg) was administered (*n* = 8/group). Blood was harvested at 24h post-APAP. We harvested serum for an analysis of alanine transaminase (ALT) (**A**) and aspartate aminotransferase (AST) (**B**). All data are expressed as mean ± SD., *n* = 8. ** *p* < 0.01, * *p* < 0.05 compared with model group; ^##^ *p* < 0.01, ^#^ *p* < 0.05 compared with control group.

In order to assess the degree of liver injury, levels of alanine transaminase (ALT) and aspartate aminotransferase (AST) in serum were detected. As presented in Figure 2, the serum ALT (A) and AST (B) levels in the model (APAP) group were elevated obviously compared to those in the control group. In contrast, BAI treatment decreased significantly the serum ALT and AST levels in the APAP-exposed mice. 

### 2.3. Effect of Baicalein on Histopathologic Changes 

**Figure 3:**
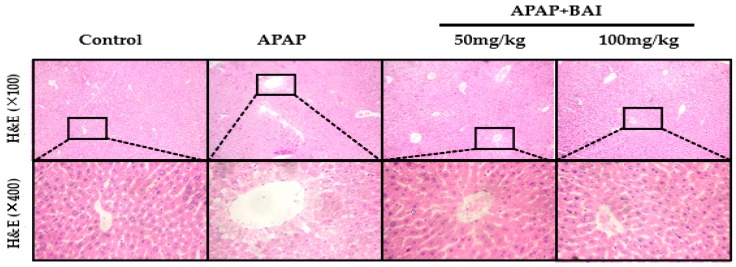
Inhibition of APAP-induced acute liver injury by BAI. Representative sections of liver stained with hematoxylin and eosin (H&E), *n* = 5. Original magnification: 100× and 400×.

As presented in Figure 3, H&E staining demonstrated that APAP induced substantial hepatic centrilobular necrosis and inflammatory cell infiltration, as well as the destruction of liver structure around the blood vessels, intrahepatic hemorrhage, and nuclear shrinkage compared with control; all the biochemical and histological changes were significantly alleviated by BAI treatment in a dose-dependent manner.

### 2.4. Effects of Baicalein on APAP-Induced Liver Oxidative Stress 

**Figure 4:**
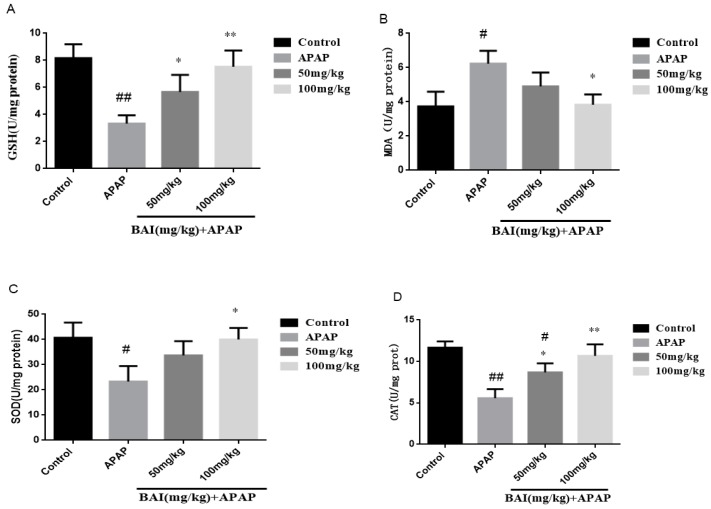
Pretreatment with BAI protected against APAP-induced liver injury: Effects of BAI on the hepatic of glutathione (GSH) (**A**), malondialdehyde (MDA) formation (**B**), superoxide dismutase (SOD) (**C**), CAT (**D**) in APAP-induced mice; values are expressed as the mean ± S.D., *n* = 8. * *p* < 0.05, ** *p* < 0.01 compared with the model group; ^#^ *p* < 0.05, ^##^ *p* < 0.01 compared with control group.

To quantify oxidative liver injury, the levels of liver superoxide dismutase (SOD), catalase (CAT), glutathione (GSH) and malondialdehyde (MDA) were measured. The results showed that compared with the control group, GSH (A), SOD (C) and CAT (D) activity in APAP group decreased significantly, liver MDA (B) level increased significantly (*p* < 0.01), baicalein (50 or 100 mg/kg) pretreatment significantly inhibited liver MDA level (*p* < 0.05; Figure 4), increased SOD, CAT and GSH activity. These results indicate that baicalein inhibits APAP induced oxidative liver injury. 

### 2.5. Effects of Baicalein on Inflammatory Cytokines Levels 

**Figure 5:**
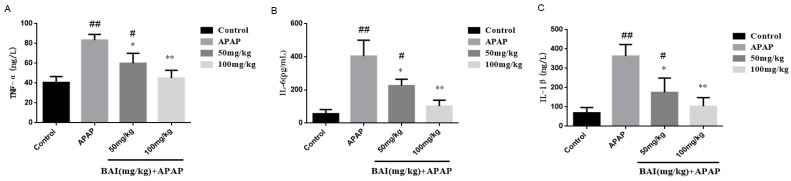
Effects of BAI on serum inflammatory responses. TNF-α (**A**), IL-6 (**B**)and IL-1β (**C**) levels from mice in each experimental group were determined by commercial kits. All data are expressed as mean ± S.D., *n* = 8. * *p* < 0.05, ** *p* < 0.01 compared with the model group; * *p* < 0.05, ** *p* < 0.01 compared with the control group.

TNF-α, IL-6 and IL-1β are the key inflammatory cytokines of fulminant liver injury induced by APAP., Serum TNF-α, IL-6 and IL-1β levels were detected by ELLSA. Meanwhile, the liver mRNA levels of TNF-α, IL-6 and IL-1β were determined by RT-PCR. The primer sequence described as Table 2. Compared with the control group, Serum levels of TNF-α, IL-1β, and IL-6 in the APAP group were significantly increased, indicating that the liver was in an over-inflammation status. When baicalein was administered, the TNF-α, IL-1β, and IL-6 decreased, indicating that baicalein had significant anti-inflammatory effects. The anti-inflammatory effect of BAI (100mg/kg) was better than BAI (50 mg/kg) (Figure 5). 

**Figure 6:**
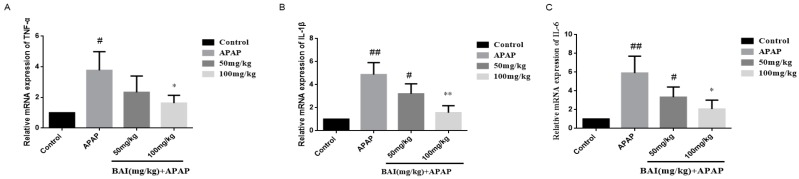
Effects of BAI on liver inflammatory responses. TNF-α (**A**), IL-6 (**B**) and IL-1β (**C**) levels from mice in each experimental group were determined by commercial kits. All data are expressed as mean ± S.D., *n* = 8. * *p* < 0.05, ** *p* < 0.01 compared with the model group; ^#^ *p* < 0.05, ^##^ *p* < 0.01 compared with control group.

As shown in Figure 6, compared with the control group, the APAP group expressed higher levels of TNF-α, IL-6, IL-1β mRNA, which were significantly reduced by BAI.

Table 2 list the primers of the real-time PCR assay used in the present work.

### 2.6. Baicalein Regulates Autophagy in Response to APAP Liver Injury

To investigate the molecular mechanisms of BAI mediated autophagy, Light Chain 3B (LC3B) Sequestosome 1 (p62), protein kinase B (AKT), and mammalian target of rapamycin (mTOR) were measured in APAP-induced liver injury. 

**Figure 7:**
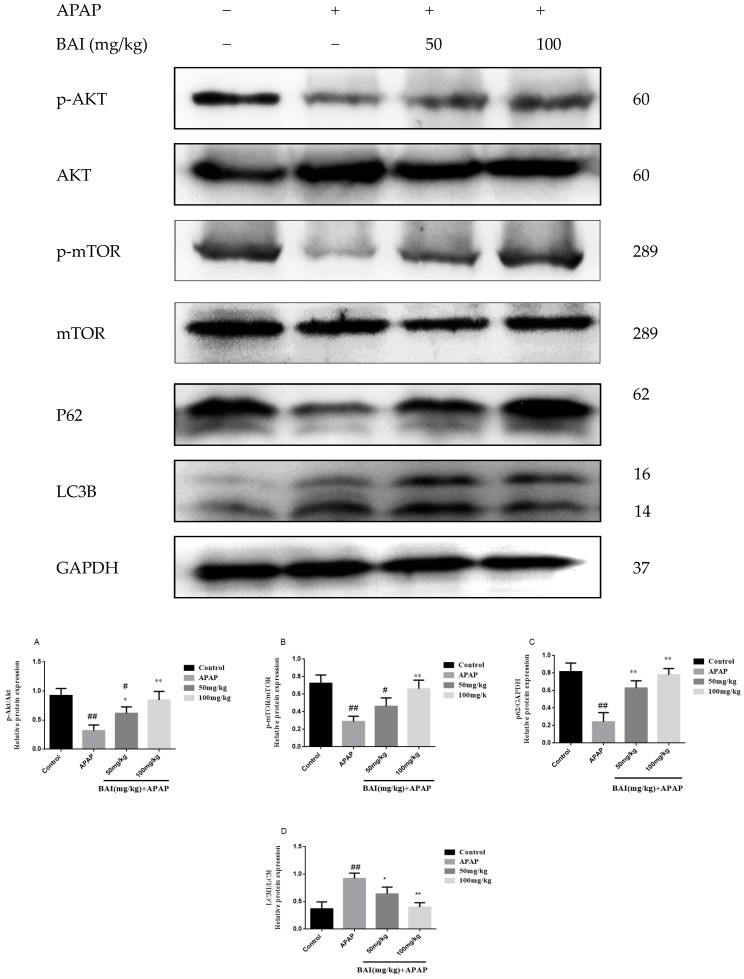
Effects of BAI on p-AKT, AKT, p-mTOR, mTOR, P62and LC3B protein expression. Protein samples were extracted from liver tissue homogenates and analyzed by western blot. Effects of BAI on APAP-induced p-AKT, AKT, p-mTOR, mTOR, P62and LC3B in liver and statistical analysis of p-AKT, AKT, p-mTOR, mTOR, P62and LC3B protein expression (**A**–**D**). Control, APAP group; 50 mg/kg, 100 mg/mg BAI group; All data are expressed as mean ± S.D., *n* = 3. ** *p* <0.01, * *p* < 0.05 compared with model group; ^##^ *p* < 0.01, ^#^ *p* < 0.05 compared with control group.

As shown in Figure 7, the increased conversion of LC3B II/I after APAP injection was decreased in a dose-dependent manner after BAI treatment. The levels of p-AKT and p-mTOR of APAP group were increased by BAI. Furthermore, BAI elevated p62 expression in comparison to the APAP group. These changes directly affected the viability of liver cells.

### 2.7. Baicalein Prevented the MAPK Pathway Activation 

**Figure 8:**
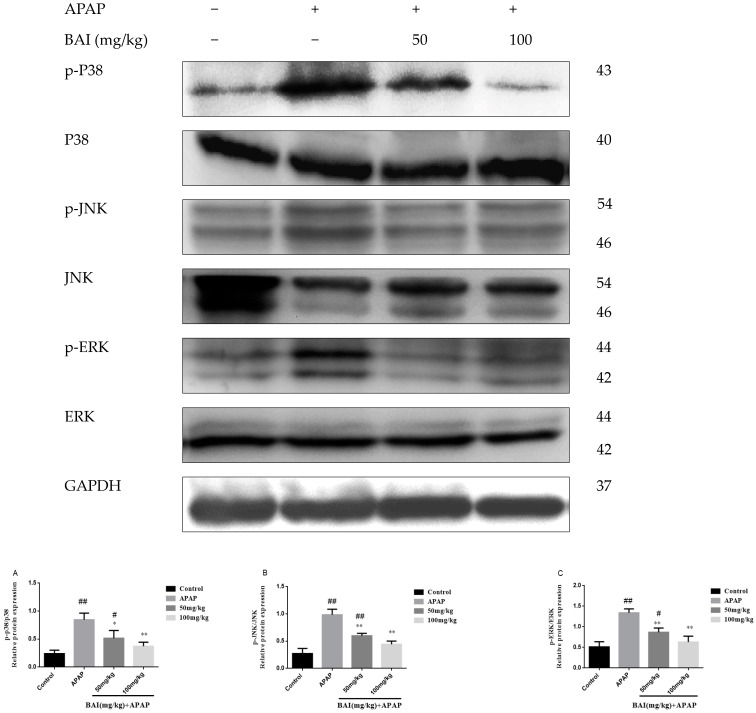
Effects of BAI on p-P38, P38, p-JNK, JNK, p-ERK and ERK protein expression. Protein samples were extracted from liver tissue homogenates and analyzed by western blot. Effects of BAI on APAP-induced p-P38, P38, p-JNK, JNK, p-ERK and ERK in liver and statistical analysis of p-P38, P38, p-JNK, JNK, JNK and ERK protein expression (**A**–**C**). Control, APAP group; 50 mg/kg, 100 mg/mg BAI group; All data are expressed as mean ± S.D. *n* = 3. * *p* < 0.05, ** *p* < 0.01 compared with the model group; ^##^ *p* <0.01, ^#^ *p* <0.05 compared with control group.

It has been reported that MAPK plays a key role in mediating APAP-induced hepatic intoxication in mice [24]. Thus, the effect of baicalein on the phosphorylation of extracellular regulated protein kinases (ERK), stress-activated protein kinase/c-Jun N-terminal kinase (JNK), and mitogen-activated protein kinases (p38 MAPK) was further evaluated. As shown in Figure 8, the levels of JNK P38 and ERK phosphorylation markedly increased after APAP treatment. In treatment with different doses of BAI (100 and 50 mg/kg). the expression of JNK, P38 and ERK phosphorylation decreased as shown in Figure 8A–D. These results are consistent with our hypothesis that BAI inhibits the MAPK signaling pathway.

### 2.8. BAI Suppressed the Expression of p-JAK2 and p-STAT3 Proteins in APAP Liver Injury

**Figure 9:**
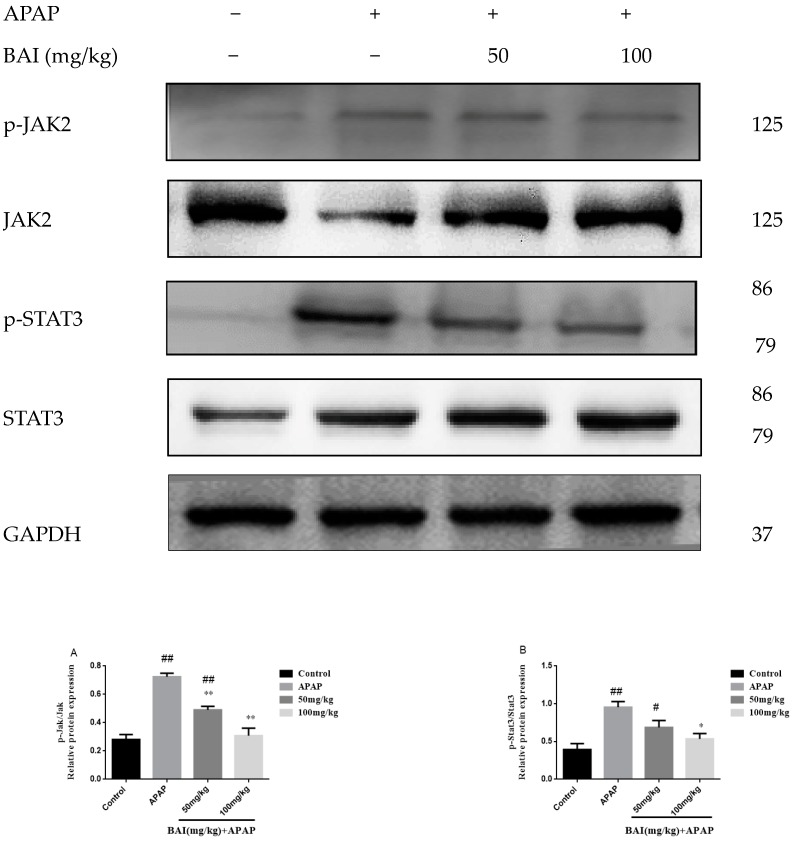
Effects of BAI on p-JAK, JAK, p-STAT3and STAT3 protein expression. Protein samples were extracted from liver tissue homogenates and analyzed by western blot. Effects of BAI on APAP-induced p-JAK2, JAK2, p-STAT3 and STAT3 in liver and statistical analysis of p-JAK2, JAK2, p-STAT3and STAT3 protein expression (**A**,**B**).Control, APAP group; 50 mg/kg, 100 mg/mg BAI group; All data are expressed as mean ± S.D. *n* = 3., ** *p* < 0.01, * *p* < 0.05 compared with model group; ^##^ *p* < 0.01, ^#^ *p* < 0.05compared with control group.

We investigated the roles of BAI in the expression of phospho-Janus kinase signal transducers 2 (p-JAK2) and phospho-Signal transducer and activator of transcription 3 (p-STAT3) proteins in APAP-induced liver injury. Although the differences were not significant, the contents of p-JAK2 and p-STAT3 (Figure 9) proteins of APAP group increased than those of the control group. However, treatment with BAI could significantly reduce p-JAK2 and p-STAT3 proteins, the expressions p-JAK2 (Figure 9A) and p-STAT3 (Figure 9B) proteins in the mice treated with BAI were significantly lower than that of the APAP group.

## 3. Discussion

The liver is more vulnerable to drugs and toxins because the metabolic rate in the liver is extremely fast. APAP overdose-caused hepatotoxicity is the most common cause of drug-induced liver failure in humans in most industrialized countries [25]. The generally accepted mechanisms of APAP-induced hepatotoxicity mainly involve oxidative stress [26], inflammation responses and autophagy of hepatocellular [27,28]. Additionally, APAP overdose could cause an increasing depletion of GSH content and accumulation of NAPQI and MDA in diseased areas, leading to morphological changes including liver metabolic dysfunction, even severe acute liver failure with a high morbidity and mortality [29,30]. Hence, it is critical to develop novel natural product to protect the liver from injury. We investigated and focused on the protective effect of BAI from APAP-induced liver damage. 

AST and ALT, as the serum hepatic biomarkers, are the two classical and main biochemical parameters of early acute liver injury, which are associated with oxidative stress [31]. In our study, we observed clearly that APAP administration markedly increased the levels of serum ALT and AST compared to the control groups, and caused severe hepatic histopathological lesions. Importantly, our experimental results suggested that serum ALT and AST levels were obviously alleviated by Baicalein in a dose-dependent manner, which suggested that baicalein exhibited a hepatoprotective effect on fulminant liver injury induced by APAP. Besides, after histopathological examination, it was confirmed that the pathological alterations induced by APAP, such as severe degeneration, centrilobular necrosis and inflammatory cell swelling, were obviously weakened by baicalein pretreatment, these results confirmed that baicalein played a significant role in the hepatoprotective effect on acute liver injury.

A great many studies demonstrate that oxidative stress is a vitally factor for hepatic dysfunction in the APAP-induced mice. APAP-induced oxidative stress can bring injury to the proteins, lipids, and DNA. Together, these events lead to cell damage [13]. To further confirm the hepatoprotective role of BAI via attenuating oxidative stress, we detected some parameters related to oxidative stress, including GSH, SOD and MDA. GSH, as a powerful antioxidant, can shield cells from oxidative damage and reduce the damage caused by APAP overdose [4]. Besides, SOD, as the pivotal ROS scavenger, can protect cells against oxidative damage [32]. MDA, as the oxidative damage biomarkers, is used to assess the oxidative stress [33]. It has been showed that the enzyme-dependent antioxidant system can cause liver dysfunction when it is overburdened. In the present study, results showed that excessive APAP caused liver tissue oxidative stress through reduced GSH level, SOD level and increased MDA content. The APAP-induced SOD depletion, GSH depletion and MDA formation were evidently reversed by Baicalein pretreatment. These experimental results indicated that the hepatoprotective activity of Baicalein might be associated with its antioxidative capacity. 

APAP-induced hepatotoxicity is also linked to inflammation because that activation of APAP metabolism leads to inflammatory cell infiltration and overexpression of inflammatory cytokines (such as TNF-α, IL-1β and IL-6), which ultimately result in inflammatory formation [29,34]. The JAK/STAT signaling pathway is intimately involved in inflammation, and the increasing number of studies have indicated that the JAK and STAT family of kinases regulate the cytokine signaling cascade [35]. JAK activation leads to activation of downstream signaling pathways, including STAT and the MAPK cascade. STAT-3exerts decisive and context-dependent functions in inflammation, tissue survival, and carcinogenesis [36]. It is worth noting that activation of STAT3 under the influence of some cytokines, including interleukin (IL)-6, IL-11, IL-13, and IL-22, has the capability to drive hepatocyte compensatory proliferation, which is a key principle of the regenerating liver [37]. A previous report by Qi et al. showed that baicalein could reduce LPS-induced inflammation via inhibiting JAK/STATs activation and ROS elevation [38]. In this study, BAI pretreatment suppressed the elevation of serum TNF-α, IL-6 and IL-1β, reduced their mRNA expressions in liver and histopathological changes, and decreased STAT3 phosphorylation in the liver tissues of APAP-treated mice. BAI could prevent APAP-induced hepatic injury via blocking of STAT3 activity. Therefore, these results demonstrated that the hepatoprotective effect of BAI was associated with its anti-inflammatory activity.

MAPK, a family of serine/threonine kinases, mainly includes JNK, ERK1/2, and p38 [39]. A massive amount of evidence has verified the crucial effect of MAPK signal pathway during APAP-treatment [40]. The activation of p38 is necessary to regulate a large number of inflammatory molecules. The ERK pathway plays an important role in the regulation of proinflammatory cytokines. JNK activation has a vital role in mediating APAP-induced hepatic damage in mice [41,42]. Recent studies also demonstrated that the protection against APAP-induced liver injury was mediated by suppressing the activation of MAPK signaling pathways [43,44]. Similarly, in this research, the elevated phosphorylation of ERK1/2, JNK, and p38 in liver tissues were observed at 24 h after APAP treatment. However, these elevated phosphorylation levels were decreased by BAI pretreatment. The result confirmed that BAI might be a potential substance to attenuate on fulminant APAP- induced hepatotoxicity in mice by mediating MAPK signaling pathway [45].

Increasing evidence suggests that enhancing autophagy may be a key hepatoprotective mechanism. The activity of autophagy is enhanced in low nutrient or inflammatory environments [46]. Commonly, autophagy is activated in response to APAP overdose in specific liver zone areas, and activation of autophagy protects against APAP hepatotoxicity [28]. LC3B and p62 are widely used as autophagy indicators to monitor autophagy process. In the process of autophagy, LC3B is an autophagic marker indicating the formation of autophagic vesicles, which could be induced from a LC3B-I form to LC3B-II form. The increase in the ratio of LC3BII/LC3BI reflects the levels of autophagic activity. The multifunctional protein p62 has been most researched as an autophagy adaptor that recruits polyubiquitinated cargo into the autophagy machinery [47,48,49]. Recent studies suggest that further inhibition of autophagy markedly exacerbated APAP-induced liver injury and protective function of autophagy is mediated through removal of the APAP-protein adducts [50,51]. Mo et al. reported that IL-22 pretreatment significantly upregulated hepatic LC3II in APAP-treated mice [52]. All these results showed that autophagy could protect the liver from APAP-induced liver injury. The AKT/mTOR signaling pathway has been corroborated to be a significant regulator of autophagy [53]. AKT is a crucial metabolic regulation enzyme that participates in the maintenance of regulating cellular metabolism to cellular energy homeostasis. It plays a protective role in an APAP-induced mouse model by promoting cell survival, oxidative stress responses, and energy generation [54,55]. mTOR is a master kinase regulating the synthesis and metabolism of protein and lipid. Pharmacological inhibition of mTOR can upregulate autophagy. mTOR is also a key kinase downstream of AKT. Activation of the AKT may result in mTOR/p70S6K pathway and subsequent autophagy activation [56]. Yim, et al. revealed that the activation of autophagy in A549 human lung carcinoma cells was due to mediation of AMPK/AKT/mTOR signaling [57].

Based on the above information, western blot analysis was performed to confirm the variation of related protein expression in the liver tissues. We found that BAI altered the status of AKT/mTOR and induced down-regulation of phosphorylation of AKT mTOR in the liver at least partially mediated by the AKT/mTOR pathway. When we further explored the role of BAI on the AKT/mTOR pathway, evidenced by the increase in autophagosome formation and LC3B II/LC3B I ratio, so BAI treatment partially inhibited the overexpression of LC3B II, accompanied by the increased protein expression of LC3B I, indicating that BAI may protect APAP-induced liver injury by modulating autophagy-related proteins.

## 4. Materials and Methods 

### 4.1. Chemicals and Reagents

Baicalein (purity > 98%) was purchased from Chengdu Pufei De Biotech Co., Ltd. (Chengdu, China). APAP was obtained from Sigma-Aldrich (St. Louis, MO, USA). The commercial assay kits for AST, ALT, MDA, GSH, SOD, CAT and hematoxylin and eosin (H&E) dye kits were purchased from Nanjing Jiancheng Bioengineering Research Institute (Nanjing, China). ELISA kits for mouse TNF-α, IL-6 and IL-1β were obtained from R&D Systems (Minneapolis, MN, USA). 

### 4.2. Experimental Setting 

Eight-week-old healthy weight-matched Kunming male mice were purchased from the Experimental Animal Holding of Jilin University with a Certificate of Quality No. SCXK-2015-0001 (Changchun, China) and along with supplies of their standard diet and adequate water. All experimental mice were strictly fed under standard conditions, maintained at controlled temperature (24–26 °C), humidity (60 ± 5%) and 12 h light/dark cycles with free access to water and food. After 1-week acclimation, Mice were randomly assigned to four groups (= 8 animals/group). The normal group (Control) and the APAP group (APAP) were administrated only 0.9% saline solution, the positive prevent group were treated with baicalein (baicalein 100, 50 mg/kg per day) for a week, APAP (350 mg/kg) was infused intraperitoneally 1 h after the last administration of positive prevent group and the APAP group. The drug was suspended in 0.05% carboxymethylcellulose sodium (CMC-Na). 24 h after APAP was infused, the mice were euthanized and harvested for blood from eyeballs and liver tissue samples. One of the liver tissues was fixed in 4% paraformaldehyde for histological analysis. Remaining the other liver tissues was stored in a deep freezer at −80 °C for further biochemical analysis. 

### 4.3. Serum ALT and AST Assays

Blood samples were centrifuged at 4 °C for 10 min at 3500× *g* to separate the serum. Alanine aminotransferase (ALT) and aspartate aminotransferase (AST) enzymatic activities of the serum were measured with commercial diagnostic assay kits (Nanjing Jiancheng Institute of Biotechnology). 

### 4.4. Histopathological Analysis 

To detect histopathological alterations, a part of the liver was carefully fixed with 10% formalin buffer, embedded in paraffin, and then sliced into 5 µm sections. The sections were stained using hematoxylin and eosin (H&E). The pathological alterations were observed using a light microscope. 

### 4.5. Measurement of Biochemical Index of GSH, SOD, CAT and MDA 

Frozen liver tissues were homogenized in iced PBS (1:9, *w*/*v*) and the homogenate was centrifuged at 3500× *g* at 4 °C for 10 min. The supernatants were collected and assayed for MDA, SOD, CAT and GSH levels using commercially available assay kits according to the manufacturer’s protocols (Nanjing Jiancheng Bioengineering Institute). 

### 4.6. TNF-α IL-6and IL-1β Analysis by ELISA

The levels of TNF-α, IL-6 and IL-1β in serum were detected by commercial ELISA kits, following the manufacturer’s instructions. Assays were performed in biological triplicates. The serum was obtained as described in “Serum AST, ALT determination”. 

### 4.7. Quantitative Real-Time PCR 

The hepatic mRNA levels of TNF-α, IL-6 and IL-1β was detected by real-time-polymerase chain reaction (RT-PCR). The total RNA of liver tissue was extracted using Ultrapure RNA Kit (Cwbiotech, Beijing, China). The RNA was reverse transcribed into cDNA with PrimeScript^TM^ RT reagent Kit with gDNA Eraser (Perfect Real Time, Takara Biomedical Technology, Beijing, China) according to the manufacturer’s instructions. RT-PCR was operated with a QTOWER3G system (Analytic Jena AG, Jena, Germany). The multiple changes between the mRNA levels in the treatment groups and the untreated group were corrected by the level of β-actin. The relative expression of mRNA was expressed by 2^−ΔΔCt^ and normalized to β-actin, an internal control gene. All target genes were repeated three times. The primers used are listed in Table 2. 

### 4.8. Western Blotting Analysis

The total protein of liver tissues was prepared by the total protein extraction reagent (BestBio, Beijing, China). The protein concentration of liver tissues was determined using the tissue BCA protein assay kit (Beyotime Biotechnology, Shanghai, China) based on the manufacturer’s protocols. The protein samples were loaded in 12% SDS-PAGE gel and transferred to a PVDF membrane. After blocked with TBST containing 5% BSA for 2 h at room temperature, the membranes were incubated overnight at 4 °C with primary antibodies including ERK (1:2000 dilution, CST, Danvers, MA, USA), p-ERK (1:1500 dilution, CST, Danvers, MA, USA), JNK (1:1500 dilution, CST, Danvers, MA, USA), p-JNK (1:1500, CST, Danvers, MA, USA), p38 MAPK (1:2000 dilution, CST, Danvers, MA, USA),p-p38 MAPK (1:1500 dilution, CST, Danvers, MA, USA), mTOR (1:1500 dilution, CST, Danvers, MA, USA), p-mTOR (1:1500 dilution, CST, Danvers, MA, USA), Akt (1:2000 dilution, CST, Danvers, MA, USA), p-Akt (1:1500 dilution, CST, Danvers, MA, USA), p62 (1:1500 dilution, CST, Danvers, MA, USA), LC3 II / I (1:1000 dilution, 4ABiotech, Beijing, China), JAK2 (1:2000 dilution, CST, Danvers, MA, USA), p-JAK2 (1:1500 dilution, CST, Danvers, MA, USA), stat3 (1:2000 dilution, CST, Danvers, MA, USA), p-stat3 (1:1500 dilution, CST, Danvers, MA, USA) and GAPDH (1:10,000 dilution, Proteintech, Chicago, IL, USA). After washing with TBST three times, the membranes were incubated for 1.5 h with the goat anti-rabbit IgG antibody (1:4000; Proteintech, Chicago, IL, USA) secondary antibodies. The membranes were visualized by ECL reagents (Tanon, Shanghai, China), before the protein bands were detected using Image Acquisition & Analysis software (analytikjena, Upland, CA, USA). 

### 4.9. Statistical Analysis 

Experimental data were expressed as mean ± SD and analyzed with GraphPad Prism 7 software package (version 5, GraphPad Software, La Jolla, CA, USA). Differences among the treatment groups were carried out by one-way ANOVA analysis of variance, followed by a post hoc comparison with the Bonferroni test. A value of *p* < 0.05 was known as statistically significant difference. 

## 5. Conclusions

In conclusion, this study confirmed that pretreatment with BAI effectively alleviated APAP-induced acute liver injury and inflammatory responses. The antioxidant activity and the anti-inflammatory activity of BAI was related to the regulation of MAPK signaling pathway, and JAK2/STAT3 signaling pathways. In addition, BAI mediated APAP-induced autophagy via AKT/mTOR signaling pathways. Therefore, all the results demonstrate that BAI may represent a potential drug for clinic therapy of chemically induced acute liver injury.

## Figures and Tables

**Table 1:** Effects of BAI on body weight and organ weight in mice.

Groups	Dosage (mg/kg)	Weight (g)		Organ Index (mg/g × 100)	
		Initial	Final	liver	Kidney
**Control**	-	29.87 ± 1.03	29.35 ± 1.22	1.32 ± 0.03	0.39 ± 0.04
**APAP+BAI**	50	29.45 ± 1.12	29.52 ± 1.21	1.33 ± 0.34	0.39 ± 0.05
**APAP+BAI**	100	29.66 ± 1.09	29.41 ± 1.13	1.36 ± 0.32	0.40 ± 0.03
**APAP**	-	29.58 ± 1.16	27.64 ± 1.01	1.47 ± 0.21	0.41 ± 0.02

Note: values are expressed as the mean ± standard deviation (S.D.), *n* = 8; ** *p* < 0.01, * *p* < 0.05 compared with model group; ^##^ *p* < 0.01, ^#^ *p* < 0.05 compared with control group.

**Table 2:** The primers of real-time PCR assay used in the present work.

Primer		Sequence	Length (bp)
**IL-1β**	F	CCCAACTGGTACATCAGCACCTC	23
	R	GACACGGATTCCATGGTGAAGTC	23
**IL-6**	F	CAAAGCCAGAGTCCTTCAGAG	21
	R	GCCACTCCTTCTGTGACTCC	20
**TNF-α**	F	TGGCCTCCCTCTCATCAG	18
	R	ACTTGGTGGTTTGCTACGAC	20
**GAPDH**	F	GTGCTATGTTGCTCTAGACTTCG	23
	R	ATGCCACAGGATTCCATACC	20
